# A queuing game theory approach to strategies for supplementing medical alliances with internet hospitals

**DOI:** 10.3389/fpubh.2025.1610722

**Published:** 2025-09-15

**Authors:** Xin Zhang, Yue Meng, Jiachun Li

**Affiliations:** Management School, Tianjin Normal University, Tianjin, China

**Keywords:** downward referral efficiency, Internet-based healthcare, referral optimization, medical alliance, two-stage queuing game

## Abstract

With large hospitals actively establishing Internet-based healthcare initiatives to facilitate the downward referral of discharged patients, it is essential to examine the conditions under which such implementations are truly beneficial. This study categorizes medical alliances (MAs) into two types: tightly integrated and loosely integrated. Utilizing queuing-game theory, we constructed a two-stage model to evaluate referral efficiency, measured by the volume of downward referrals from tertiary hospitals and the effort levels exerted by community hospitals. By comparing MAs with and without supplementary internet hospitals, we identify the circumstances in which hospital-established internet hospitals enhance patient referrals. The findings indicate that within tightly integrated MAs, the referral volume increases with the potential patient arrival rate. When the cost coefficient of internet hospitals is low, MAs that incorporate internet hospitals demonstrate both higher referral volumes and increased effort, with profits favored by Internet-based healthcare under low arrival rates. In loosely integrated MAs, effort levels exhibit a similar pattern while referral volume depends heavily on the revenue-sharing ratio of the internet hospital. Specifically, referral volume increases when the ratio is low, arrival rates are high, and cost coefficients remain low. Conversely, at high revenue-sharing ratios, referral volumes rise regardless of the cost coefficient, provided arrival rates are either low or high. For tertiary hospitals, profits are higher with Internet-based healthcare only under low arrival rates when the revenue-sharing ratio is low; this threshold declines as the cost coefficient increases. When the ratio is high, digital healthcare consistently yields higher profits. For community hospitals, a low ratio leads to higher profits only within a moderate range of arrival rates—a range that narrows with rising cost coefficients. Under high ratios, profitability improves only at low arrival rates and increases alongside the cost coefficient.

## 1 Introduction

With rapid socioeconomic development and continuous improvement in living standards, the demand for healthcare services is rising. Although the overall supply of medical resources increases each year, challenges such as high costs, limited access, uneven distribution, and underutilization of services persist. Consequently, the government has promoted a hierarchical healthcare system, encouraging collaboration and division of labor among medical institutions at different levels and shifting general diagnoses and treatments to primary care settings. However, factors such as conflicting hospital interests and limited service capacity of primary care facilities impede the effective implementation of two-way referrals. To refine and popularize the hierarchical diagnosis and treatment system, promote downward patient referrals, and alleviate the imbalance between healthcare supply and demand, China has actively encouraged the development of medical alliances (MAs) since 2009. By the end of 2023, over 18,000 MAs have been established nationwide, enabling 14.722 million downward patient referrals—a 29.9% increase over the previous year. The issue of MA referral efficiency is crucial for the implementation of hierarchical treatment policies, yet it has received limited scholarly attention to date.

At the same time, ongoing advancements in information technology have given rise to new models of healthcare delivery. Among them, Internet-based healthcare has rapidly expanded—especially under normalized epidemic prevention and control—easing the burden on central hospitals. This model provides robust technological support for initial consultations within MA community clinics, chronic disease management, prescription renewals, and two-way referrals. As such, innovative collaborations incorporating Internet-based healthcare contribute meaningfully to the development of a hierarchical treatment system. While some scholars have explored the integration of Internet-based healthcare and MAs, critical questions remain—particularly whether such integration genuinely benefits hierarchical healthcare delivery. Therefore, examining the complementary role and operational mechanisms of Internet-based healthcare within MAs is essential for advancing the implementation of hierarchical treatment and enhancing downward referral effectiveness.

Building upon this, this study aims to identify the optimal strategies for supplementing MAs with Internet-based healthcare. Initially, existing literature on the collaboration between internet hospitals and MAs, patient referrals, and MA queuing service systems will be reviewed and synthesized. Drawing from this body of research, we develop a two-stage queuing-game model to capture the dynamic interactions among tertiary A-level hospitals, community hospitals, internet hospitals, and patients. Referral efficiency is quantified by the volume of downward referrals from tertiary hospitals and the effort level of community hospitals. By optimizing the model and analyzing objective functions and equilibrium solutions under varying conditions, we compare the referral efficiency of MAs with and without internet hospital support. This comparison allows us to identify the conditions under which hospital-established internet hospitals effectively facilitate patient referrals.

## 2 Literature review

The relevant literature for this study spans four key domains: Internet-based healthcare, medical alliances (MAs), hierarchical diagnosis and treatment, and queuing service systems.

In recent years, research on internet hospitals has primarily centered on innovations in telemedicine and remote monitoring technologies, exploring how these tools can enhance healthcare efficiency and improve patient management and disease monitoring. Davis et al. noted that remote medical services have entered a phase of stable growth, with Internet-based healthcare effectively facilitating remote diagnosis, monitoring, and doctor–patient communication through technologies such as video conferencing, sensors, and wearable devices (1). Ahmad et al. explored the potential opportunities and adaptive challenges of blockchain technology in telemedicine and remote healthcare fields. Blockchain plays a critical role in strengthening information security and privacy protection, while also enhancing the transparency of business operations. By ensuring the immutability and traceability of data, it enables effective monitoring of fraudulent activities such as false patient insurance claims and physician credential verification (2). As research into the behavior of Internet healthcare users deepens, the significance of satisfaction evaluation has become increasingly apparent. Singh et al. conducted empirical studies to examine the differing expectations of patients and doctors within the emerging Internet healthcare device ecosystem. Their findings revealed that device usability significantly boosted patient satisfaction, whereas the increase in doctor satisfaction was comparatively modest (3). Wang et al. employed Python crawlers to extract text content from Internet healthcare platforms and applied natural language processing (NLP) techniques to develop an information quality evaluation index system (4). Some scholars focus on the regulatory issues of Internet-based healthcare. Marelli et al. pointed out that the rapid advancement of digital healthcare has outpaced the adaptability of existing data governance systems, necessitating the construction of more flexible and rapidly responsive regulatory policies (5). Xanthidou et al. conducted an in-depth study on the application of electronic health records, identifying several challenges related to data access rights, security regulations, and authorization protocols. In summary, existing research on Internet-based healthcare predominantly addresses its service benefits, user adoption, and regulatory challenges. However, studies examining the collaboration between Internet healthcare and MAs remain limited. This study bridges that gap by integrating Internet-based healthcare with MAs, investigating the effectiveness of Internet healthcare as a supplementary channel to enhance downward patient referrals within MAs, thereby expanding the research landscape of Internet-based healthcare (6).

Another stream of literature pertains to MAs. Due to differences in social structures and healthcare systems worldwide, no universally consistent concept functionally or structurally equivalent to the “Medical Alliance” has yet emerged. The closest international counterpart is integrated healthcare services, which emphasize collaborative operations and comprehensive integration among medical institutions. This approach aims to optimize the allocation of medical resources while simultaneously improving service fairness and accessibility. Some scholars have explored the development of integrated healthcare services. For example, Bernard argued that rapid advances in Internet technology have positioned telemedicine services as a vital tool for optimizing medical resource allocation and management, thereby significantly enhancing healthcare efficiency and accessibility (7). Baxter et al. developed an integrated healthcare service model grounded in evidence-based policy and assessed its effectiveness in improving patient service quality, optimizing labor structure, streamlining work processes, maintaining financial stability, and strengthening management integration (8). Some researchers concentrate on model innovation and practical application. Li et al. examined the development of a tightly integrated medical alliance at the Second People's Hospital of Guangdong Province, identifying innovative approaches and categorizing the construction of tightly integrated MAs into three distinct models: business-led, participatory advisory, and menu-customized (9). Some scholars focus on the effectiveness evaluation of MAs. He et al., using urban MAs as their research subject and employing the difference-in-differences method, found that MAs significantly enhance the medical service capacity, clinical standards, and administrative efficiency of secondary and lower-level hospitals within the alliance. These improvements are driven by strengthened technology spillover and management coordination effects, which also contribute to more harmonious doctor–patient relationships (10). Currently, research on the collaboration between MAs and Internet-based healthcare is limited. Most studies concentrate on the application of MA and Internet healthcare collaboration in promoting the hierarchical diagnosis and treatment system (reviewed in the section on hierarchical diagnosis and treatment). A smaller subset of research examines the informatization of MAs. For example, Xu et al., in addressing the uneven distribution of medical resources among hospitals, emphasized the construction and application of information platforms. Their work actively explores the role of “Internet+” in advancing MA development, with the goal of achieving vertical integration of medical resources and strengthening primary healthcare service capacity (11). Wang et al. pointed out that insufficient interoperability of information systems within MAs is a major obstacle, highlighting the urgent need to strengthen and optimize Internet platform infrastructure while also improving public awareness and acceptance (12). In summary, both domestic and international scholars have examined the construction and practical application of MAs, yet research on their collaboration with Internet hospitals remains limited, primarily focusing on the informatization of MAs. Few scholars have explored the selection problem of Internet hospitals within MAs, but the research assumes hospital scale as the premise. This paper uses the downward referral efficiency of MAs promoted by Internet-based healthcare as the basis to explore the selection of hospital-owned Internet hospitals by MAs.

Due to differences in healthcare system structures and policy environments across countries, the concept of hierarchical diagnosis and treatment has not been formally established abroad. Its closest counterpart is the three-tier healthcare service system. Nonetheless, there is broad consensus within the medical community on the necessity of implementing hierarchical diagnosis and treatment systems. Greenfield et al. found that primary healthcare services are highly effective in controlling medical costs and play a key role in easing the burden on medical resources. However, the practical implementation of a hierarchical diagnosis and treatment system remains complex, requiring large-scale system coordination and being influenced by a wide range of factors (13). Zhao evaluated the impact of MA policies on hierarchical diagnosis and treatment from the perspective of medical staff, finding that the institutional level significantly influences evaluation outcomes. Primary medical institutions, in particular, tend to focus on policy formulation and implementation to secure associated benefits (14). Some scholars have examined the optimization of MA resource allocation. Liu et al. and Wang et al. investigated the problem of determining optimal referral rates in a two-tier service system—comprising tertiary hospitals and community hospitals—by constructing sequential game models under both centralized and decentralized decision-making scenarios (15, 16). Other scholars focus on research concerning the hierarchical diagnosis and treatment system under the MA framework. Ma et al. believe that MAs are important carriers of the hierarchical diagnosis and treatment system. Based on practical observations, they identified several challenges, including weak incentives for downward referrals, limited primary care capacity, and difficulties in coordinating the interests of MA member institutions. To address these issues, they proposed measures such as improving referral procedures, strengthening triage guidance, leveraging the strengths of primary care, and enhancing the overall service capabilities of lower level institutions (17). Some scholars have explored the construction of hierarchical diagnosis and treatment systems and the development of new models from the perspective of “Internet+”. Heng et al. proposed that “Internet+” hierarchical diagnosis and treatment utilizes informatization and intelligent means to assist MAs in efficiently integrating regional medical resources. Leveraging localized advantages, it can maximally integrate medical resources and improve diagnostic efficiency (18). In recent years, many scholars have realized the important role of the hierarchical diagnosis and treatment system in alleviating the problem of unreasonable medical resource allocation and have studied patient downward referrals. Wang et al. using simulation optimization techniques, verified that implementing downward referral strategies can effectively alleviate resource constraints in higher-level hospitals and improve resource utilization in lower-level hospitals (19). In addition to constructing queuing optimization models to find optimal referral strategies, some scholars consider the non-cooperative competitive relationship between general hospitals and primary care hospitals, analyzing the game interactions among multiple participants. Yao et al. constructed a three-party evolutionary game model involving medical insurance departments, hospitals, and critically ill patients in tertiary hospitals. They explored the evolutionary paths and influencing factors of hospital and patient strategies under different government coordination strategies, concluding that the government holds a coordinating C-level position in advancing hierarchical diagnosis and treatment (20). Most of the literature reviewed examines Internet-based healthcare as a supplementary pathway for MAs, highlighting its potential and effectiveness in advancing the hierarchical diagnosis and treatment system. However, its actual impact remains contested, and diverse “Internet+MA” models have been proposed and studied. Some scholars conduct quantitative research on optimizing medical resource allocation in the hierarchical diagnosis and treatment system, but their studies do not incorporate Internet-based healthcare. This paper integrates Internet-based healthcare with MAs, employing quantitative mathematical modeling to examine the effectiveness of Internet healthcare as a supplementary channel for promoting patient downward referrals, thereby expanding the research frontier on their collaboration.

Furthermore, queuing theory provides a solid methodological foundation for optimizing resource allocation and utilization, facilitating rigorous and precise analysis of complex real-world queuing challenges. International scholars have applied queuing theory principles and methods to develop scientific optimization solutions for hospital operations. Tyagi et al. aiming to reduce patient waiting times and improve medical resource utilization, applied queuing theory to optimize the number of servers in a healthcare service system (21). Ala et al., through case studies applying queuing theory, utilized mixed-integer linear programming (MILP) and approximate solution algorithms (ASA) to tackle supply chain network design and patient scheduling problems in healthcare (22). Wang Wenjuan et al. established an integrated queuing and game model for a referral system, exploring how the government can incline patients toward initial community consultations by configuring hospital scales and adjusting medical service prices (23). Yu used queuing theory to construct a game model involving patients, tertiary A-level hospitals, and community hospitals, investigating effective subsidy strategies to guide the downward allocation of medical resources (24). This study similarly employs queuing theory to analyze the downward referral efficiency of MAs. However, unlike previous studies, it incorporates Internet-based healthcare into the model, examining its impact on stakeholders, decision-making stages, and processes within MAs under various cooperation modes following the integration of Internet hospitals, thereby expanding the scope of existing research.

In summary, while existing literature offers valuable insights, a comprehensive review reveals notable gaps. Building on prior research, this paper develops a queuing-game model to evaluate the effectiveness of hospital-owned Internet hospitals in supplementing MAs and facilitating patient downward referrals. Furthermore, it advances the analysis by categorizing MAs by type and exploring optimal strategies for integrating Internet hospitals across different MA models.

## 3 Model development

This study considers a two-tier healthcare service system comprising one tertiary A-level hospital (hereinafter referred to as the tertiary hospital) and one community hospital. Patients initially visit the tertiary hospital, which refers those with milder conditions to the community hospital, while retaining severe cases for further treatment. The tertiary hospital offers more specialized services at higher costs, whereas the community hospital treats only minor cases at lower costs. With the introduction of Internet-based healthcare, the tertiary hospital can enhance the community hospital's service capacity through online support. This not only helps free up its own resources and accommodate more patients but also promotes more efficient use of community hospital resources, thereby improving the overall performance of the healthcare system.

Patients arrive at the tertiary hospital according to a Poisson distribution with a potential arrival rate of Λ. Based on their perceived utility U, patients decide whether to enter the system. Those who ultimately receive treatment are termed the effective arrival rate, denoted by λ_*e*_. For instance, if 25 patients seek treatment (indicating a potential arrival rate of 25), but 5 patients opt out due to factors like long wait times, the remaining 20 constitute the effective arrival rate. Patient conditions are heterogeneous and represented by φ, where a higher φ, indicates a more severe condition and increased likelihood of remaining in the tertiary hospital for follow-up care. The community hospital can only provide follow-up services for patients with φ ∈ [0, φ_0_], Thus, the downward referral quantity to the community hospital is $q=\int_{0}^{\varphi_{0}}f\left(\varphi \right)d\varphi$. That is, the clinical transfer criteria established by tertiary hospitals.

Assume the service capacity of the tertiary hospital is constant at *v*_*H*_. If a patient receives complete treatment at the tertiary hospital, the total service time follows an exponential distribution with mean $\frac{1}{v_{H}}$, Let $\frac{\theta }{v_{H}}$ and $\frac{1-\theta }{v_{H}}$ represent the time for surgery and subsequent recovery, respectively. θ denotes the proportion of time allocated to preliminary surgical treatment at tertiary hospitals relative to total treatment time. When θ = 0.4, this indicates that surgical procedures constitute 40% of the total treatment duration. After completing surgical treatment at the tertiary hospital, a patient has a probability $\frac{q}{\lambda_{e}}$ of being referred to the community hospital and a probability $1-\frac{q}{\lambda_{e}}$ of remaining in the tertiary hospital. Assume the cost of treatment is fully covered by the Medical Insurance Bureau. Under the DRG-based payment system, the tertiary hospital earns a revenue of *g*_1_ and incurs a cost of *C*_1*H*_ for providing initial surgical treatment services per patient. For providing subsequent recovery services, the revenue is *g*_2_ and the cost is *C*_2*H*_.

Assume the community hospital's service capacity is constant at *v*_*L*_, and it serves referred patients. Referred patients arrive at the community hospital following a Poisson distribution with rate *q*. Drawing lessons from Shenzhen's capitation-based payment model for primary care clinics, the community hospital earns a revenue of *g*_*L*_ and incurs a cost of *C*_*L*_ for providing subsequent recovery services per patient. Community hospital require corresponding manpower, technology and other inputs to provide services, that is the effort level *e*. The effort-related cost required for the community hospital to enhance its service level is $C_{E}=C_{e}e^{2}$, which increases with the effort level *e*. When Internet-based healthcare is introduced, the community hospital's effort-related cost becomes $C_{E}=\frac{C_{e}e^{2}}{\gamma }$, where γ represents the service level provided by the Internet hospital; the unit effort-related cost decreases as γ increases. When the community hospital fails to treat a patient due to insufficient capacity, it incurs a penalty cost $M=\frac{mq^{2}}{e}$. This penalty cost increases with the referral quantity *q* and decreases with the effort level *e*. In a tightly integrated MA, the entire MA bears the penalty cost. In a loosely integrated MA, the cost is shared proportionally, with the tertiary hospital bearing α*M*and the community hospital bearing (1−α)*M*, where α is an exogenous parameter.

Assume the Internet hospital has sufficient service capacity. Based on the current operations of the Bohe Doctor Platform, the initial establishment phase requires investment in development costs, with technical and operational expenditures increasing as more personalized services are offered. The construction and operation of the Internet hospital incur a corresponding technological cost $C_{p}=d_{\gamma }^{2}$, where *d*is the marginal cost. Drawing on the revenue-sharing mechanism implemented by Shanghai Putuo District Central Hospital, community hospitals receive reimbursements from health insurance funds for accepting rehabilitative patients, while the internet platform retains 10% as a technical support fee. The Internet hospital adopts a revenue-sharing model to distribute the revenue obtained from treating referred patients at the community hospital, *g*_*s*_ = β*g*_*L*_.

This study examines tightly integrated and loosely integrated MAs before and after the introduction of hospital-owned Internet hospitals.

Scenario 1: Tightly integrated MA without the Internet hospital. The MA determines the optimal referral quantity *q* (from the tertiary hospital) and effort level *e* (of the community hospital) based on maximizing overall profit. Patients then decide whether to seek treatment based on their individual utility.

Scenario 2: Loosely integrated MA without the Internet hospital. The tertiary hospital first decides the referral quantity *q* based on maximizing its own profit. Then, the community hospital determines its effort level *e*. Finally, patients decide whether to enter the system.

Scenario 3: Tightly integrated MA with the hospital-owned Internet hospital. The MA determines the optimal referral quantity *q*, community hospital effort level *e*, and Internet hospital service level γ based on maximizing overall profit. Patients then decide whether to seek treatment.

Scenario 4: Loosely integrated MA with the hospital-owned Internet hospital. The tertiary hospital first determines the referral quantity *q* and Internet hospital service level γ based on maximizing its own profit. Then, the community hospital determines its effort level *e*. Finally, patients decide whether to enter the system.

### 3.1 Patient treatment choice decision model

Let U represent the net utility for a patient entering the healthcare system. Assuming treatment costs are fully covered by medical insurance, if a patient enters the tertiary hospital, their expected waiting time is *E*(ε). The net utility can be expressed as:


(3.1) $$\begin{array}{l} U=R-bE\left(\varepsilon \right) \end{array}$$

where $E\left(\varepsilon \right)=\frac{\lambda_{e}\theta^{2}+\left(1-\theta \right)\left(\lambda_{e}-q\right)}{v_{H}\left(v_{H}-\lambda_{e}+q\left(1-\theta \right)\right)}$, is the patient's perceived value of treatment, and b is the cost per unit of waiting time. Patients choose to enter the tertiary hospital if *U* ≥ 0.

Theorem 3.1:The patient's equilibrium arrival rate λ_*e*_ depends on the potential arrival rate Λ and the tertiary hospital's referral quantity *q*.

The equilibrium arrival rate is:


(3.2) $$\begin{array}{l} \lambda_{e}=\{\begin{array}{c} \lambda_{0},0<q<\frac{\left(b+b\left(1-\theta \right)\theta +Rv_{H}\right)\Lambda -Rv_{H}^{2}}{\left(1-\theta \right)\left(b+Rv_{H}\right)}\\ \Lambda ,\frac{\left(b+b\left(1-\theta \right)\theta +Rv_{H}\right)\Lambda -Rv_{H}^{2}}{\left(1-\theta \right)\left(b+Rv_{H}\right)}\leq q\leq \Lambda \end{array}\\ \lambda_{0}=\frac{Rv_{H}^{2}+q\left(1-\theta \right)\left(b+Rv_{H}\right)}{b\theta^{2}+b\left(1-\theta \right)+Rv_{H}} \end{array}$$

Given a constant potential arrival rate Λ, when the tertiary hospital's referral quantity is small, more patients utilize the tertiary hospital's resources for subsequent recovery, leading to longer waiting times due to insufficient capacity. Thus, the equilibrium arrival rate λ_*e*_ increases with the referral quantity *q*. Once *q* exceeds a certain threshold, the tertiary hospital's capacity is sufficient to handle all incoming patients' initial treatment services, so all potential patients enter the system, and λ_*e*_ no longer changes, becoming equal to Λ.

### 3.2 MA revenue model without internet hospital introduction

Tightly integrated MA: The MA obtains revenue from services provided by both hospitals but bears the penalty cost and the community hospital's effort-related cost collectively. The MA determines the optimal referral quantity *q* and effort level *e* to maximize overall profit.

Total profit function:


(3.3) $$\begin{array}{l} \pi_{T}=\left(g_{1}-C_{1H}\right)\lambda_{\text{e}}+\left(g_{2}-C_{2H}\right)\left(\lambda_{\text{e}}-q\right)+\left(g_{L}-C_{L}\right)\\ \;q-C_{E}\left(e\right)q-M\left(q,e\right) \end{array}$$

Theorem 3.2: In a tightly integrated MA, there exist optimal *q* and *e* that maximize profit. The optimal effort level for the community hospital is $e_{1}=3\sqrt{\frac{mq}{2C_{e}}}$. The optimal referral quantity *q* depends on the potential arrival rate Λ :


$$\begin{array}{l} q^{C}=\{\begin{array}{l} \Lambda ,\;0<\Lambda \leq q_{2}\\ \begin{array}{l} q_{\text{2}},q_{2}<\Lambda \leq \frac{Rv_{H}^{2}+q_{2}\left(1-\theta \right)\left(b+Rv_{H}\right)}{b\theta^{2}+b\left(1-\theta \right)+Rv_{H}}\\ q_{0},\frac{Rv_{H}^{2}+q_{2}\left(1-\theta \right)\left(b+Rv_{H}\right)}{b\theta^{2}+b\left(1-\theta \right)+Rv_{H}}<\Lambda \leq \frac{Rv_{H}^{2}+q_{1}\left(1-\theta \right)\left(b+Rv_{H}\right)}{b\theta^{2}+b\left(1-\theta \right)+Rv_{H}} \end{array}\\ q_{1},\Lambda \;>\frac{Rv_{H}^{2}+q_{1}\left(1-\theta \right)\left(b+Rv_{H}\right)}{b\theta^{2}+b\left(1-\theta \right)+Rv_{H}} \end{array} \end{array}$$

Loosely integrated MA: Hospitals make decisions separately. The tertiary hospital maximizes its profit to determine *q*, and the community hospital maximizes its profit to determine *e*, considering their respective shares of the penalty cost.

Tertiary Hospital Profit:


(3.4) $$\begin{array}{l} \pi_{H}=\left(g_{1}-C_{1H}\right)\lambda_{\text{e}}+\left(g_{2}-C_{2H}\right)\left(\lambda_{\text{e}}-q\right)-\alpha M\left(q,e\right) \end{array}$$

Community Hospital Profit:


(3.5) $$\begin{array}{l} \pi_{L}=\left(g_{L}-C_{L}\right)q-C_{E}\left(e\right)q-\left(1-\alpha \right)M\left(q,e\right) \end{array}$$

Theorem 3.3: In a loosely integrated MA, optimal *q* and *e* exist. The optimal effort level is $e_{2}=\sqrt[{3}]{\frac{\left(1-\alpha \right)mq}{2C_{e}}}$. The optimal referral quantity *q* D depends on Λ :


$$\begin{array}{l} q^{D}=\{\begin{array}{c} q_{0},\Lambda \leq \frac{Rv_{H}^{2}+q_{3}\left(1-\theta \right)\left(b+Rv_{H}\right)}{b\theta^{2}+b\left(1-\theta \right)+Rv_{H}}\\ q_{3},\Lambda \;>\frac{Rv_{H}^{2}+q_{3}\left(1-\theta \right)\left(b+Rv_{H}\right)}{b\theta^{2}+b\left(1-\theta \right)+Rv_{H}} \end{array} \end{array}$$

### 3.3 MA revenue model with internet hospital introduction

Tightly integrated MA: The MA earns revenue, facilitates referrals via the Internet hospital, and helps the community hospital improve its service level. The MA bears the penalty cost, effort-related cost, and Internet hospital cost. It determines optimal *q*, *e*, and γ.

Total Profit Function:


(3.6) $$\begin{array}{l} \pi_{T}=\left(g_{1}-C_{1H}\right)\lambda_{e}+\left(g_{2}-C_{2H}\right)\left(\lambda_{e}-q\right)+\left(g_{L}-C_{L}\right)q\\ \;-C_{E}q-M-C_{p} \end{array}$$

Theorem 3.4: In a tightly integrated MA with an Internet hospital, optimal *q*, *e*, and γ exist. The optimal effort level is $e_{3}=\sqrt[{3}]{\frac{\gamma mq}{2C_{e}}}$. The optimal referral quantity *q*^*C*^ depends on Λ. The maximum profit:


$$\begin{array}{l} q^{C}=\{\begin{array}{l} \Lambda ,0<\Lambda \leq q_{6}\\ \begin{array}{l} q_{\text{6}},q_{6}<\Lambda \leq \frac{Rv_{H}^{2}+q_{6}\left(1-\theta \right)\left(b+Rv_{H}\right)}{b\theta^{2}+b\left(1-\theta \right)+Rv_{H}}\\ q_{0},\frac{Rv_{H}^{2}+q_{6}\left(1-\theta \right)\left(b+Rv_{H}\right)}{b\theta^{2}+b\left(1-\theta \right)+Rv_{H}}<\Lambda \leq \frac{Rv_{H}^{2}+q_{5}\left(1-\theta \right)\left(b+Rv_{H}\right)}{b\theta^{2}+b\left(1-\theta \right)+Rv_{H}} \end{array}\\ q_{5},\Lambda >\frac{Rv_{H}^{2}+q_{5}\left(1-\theta \right)\left(b+Rv_{H}\right)}{b\theta^{2}+b\left(1-\theta \right)+Rv_{H}} \end{array} \end{array}$$

Loosely integrated MA: Hospitals decide separately. The tertiary hospital determines *q* and γ. The community hospital determines *e*. The tertiary hospital shares β of the community hospital's revenue *g*_*L*_ and both share the penalty cost α*M*and (1−α)*M*, respectively.

Tertiary Hospital Profit:


(3.7) $$\begin{array}{l} \pi_{H}=\left(g_{1}-C_{1H}\right)\lambda_{\text{e}}+\left(g_{2}-C_{2H}\right)\left(\lambda_{\text{e}}-q\right)+\beta g_{L}q\\ \;-\alpha M\left(q,e\right)-C_{p}\left(\gamma \right) \end{array}$$

Community Hospital Profit:


(3.8) $$\begin{array}{l} \pi_{L}=\left(\left(1-\beta \right)g_{L}-C_{L}\right)q-C_{E}\left(e\right)\\ \;q-\left(1-\alpha \right)M\left(q,e\right) \end{array}$$

Theorem 3.5: In a loosely integrated MA with an Internet hospital, optimal *q*, *e*, and γ exist. The optimal effort level is $e_{4}=\sqrt[{3}]{\frac{\left(1-\alpha \right)\gamma mq}{2C_{e}}}$. The optimal referral quantity *q*^*D*^' depends on Λ and the revenue sharing factor β :

If $\beta \geq \frac{g_{2}-C_{2H}}{g_{L}}$:


$$q^{D}=\{\begin{array}{l} \Lambda ,\;0<\Lambda \leq q_{8}\\ \begin{array}{l} q_{8},q_{8}<\Lambda \leq \frac{Rv_{H}^{2}+q_{8}(1-\theta )(b+Rv_{H})}{b\theta^{2}+b(1-\theta )+Rv_{H}}\\ q_{0},\frac{Rv_{H}^{2}+q_{8}(1-\theta )(b+Rv_{H})}{b\theta^{2}+b(1-\theta )+Rv_{H}}<\Lambda \leq \frac{Rv_{H}^{2}+q_{7}(1-\theta )(b+Rv_{H})}{b\theta^{2}+b(1-\theta )+Rv_{H}} \end{array}\\ q_{7},\Lambda \;>\frac{Rv_{H}^{2}+q_{7}(1-\theta )(b+Rv_{H})}{b\theta^{2}+b(1-\theta )+Rv_{H}} \end{array}$$

If $0<\beta <\frac{g_{2}-C_{2H}}{g_{L}}$:


$$q^{D}=\{\begin{array}{c} q_{0},\Lambda \leq \frac{Rv_{H}^{2}+q_{7}\left(1-\theta \right)\left(b+Rv_{H}\right)}{b\theta^{2}+b\left(1-\theta \right)+Rv_{H}}\\ q_{7},\Lambda >\frac{Rv_{H}^{2}+q_{7}\left(1-\theta \right)\left(b+Rv_{H}\right)}{b\theta^{2}+b\left(1-\theta \right)+Rv_{H}} \end{array}$$

## 4 Comparative analysis and numerical simulation

(Using MATLAB simulation with parameters: *g*_1_ = 8, *C*_1*H*_ = 6, *g*_2_ = 6, *C*_2*H*_, *g*_*L*_ = 5, *C*_*L*_ = 1, *C*_*e*_ = 1, *m* = 0.3, *R* = 10, *b* = 2, *v*_*H*_ = 5, θ = 0.4.α = 0.5 for loosely integrated cases).

### 4.1 Comparative analysis of optimal decisions in tightly integrated MA

Proposition 1: The referral quantity in a tightly integrated MA is influenced by the Internet hospital's cost coefficient *d* and the potential patient arrival rate λ.

(1) Referral quantity increases withΛ. When Λ is very small or very large (such that some potential patients do not enter), the Internet hospital cannot increase referrals.
(2) There exists a threshold d^*^. If 0 < *d* < d^*^, introducing a hospital-owned Internet hospital leads to a referral quantity not lower than without it. If *d* > d^*^, the referral quantity is higher without the Internet hospital.

**Figure 1:**
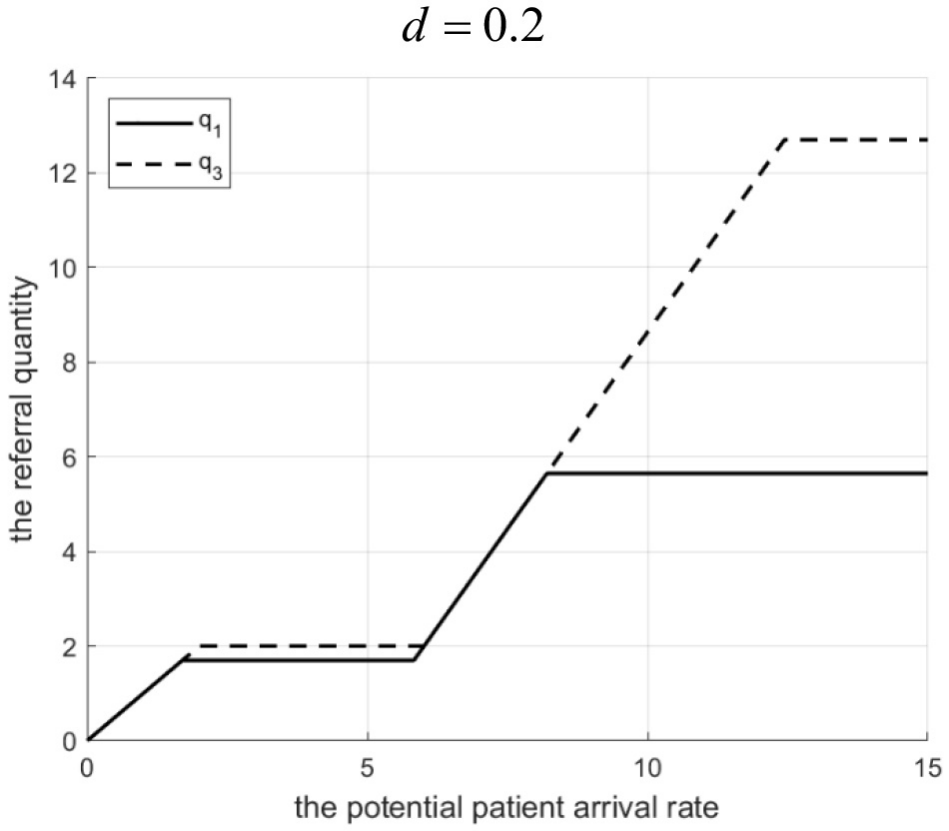
Referral quantity in a tightly integrated MA before and after the introduction of internet medical care (*d* = 0.2).

**Figure 2:**
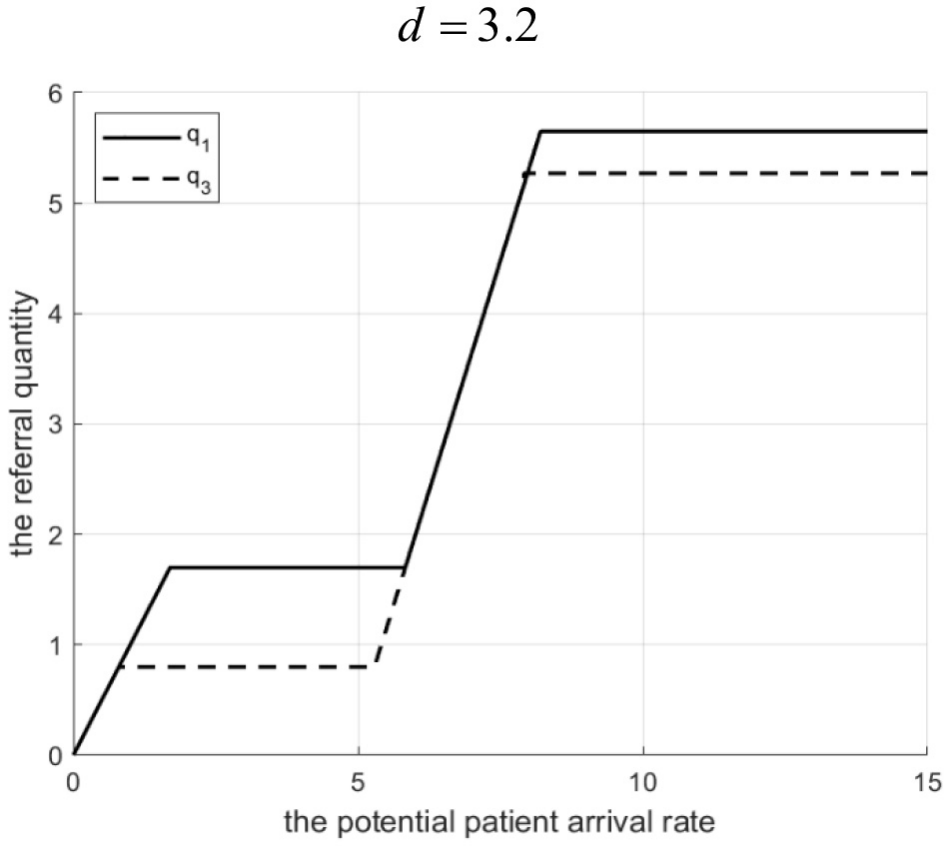
Referral quantity in a tightly integrated MA before and after the introduction of internet medical care (*d* = 3.2).

Rationale and Simulation (Figure 1 *d* = 0.2, Figure 2 *d* = 3.2): When Λ is small, MA resources are ample, minimizing penalty cost dictates low referrals. When Λ is large but some don't enter, raising referrals increases penalty > benefit, so referrals aren't increased. As Λ grows in the mid-range, treatment benefits exceed penalty costs, encouraging higher referrals. The Internet hospital enhances community capacity, raising the referral threshold. Low-cost *d* means referral benefits cover Internet costs, encouraging higher service levels (γ) and thus higher referrals (*q*_3_ > *q*_1_ in Figure 1). High-cost *d* makes Internet operation costly, leading to lower γ, reduced community effort, and lower referrals to minimize penalties (*q*_3_ < *q*_1_ in Figure 2 where *q*_1_ and *q*_3_ denote the downward referral volumes before and after implementing Internet-based healthcare in tightly integrated MA, respectively).

Proposition 2: For a given referral quantity, the service effort of the community hospital in a tightly integrated MA is influenced by the Internet hospital's cost coefficient *d*. There exists *d*_1_. If, effort is higher with the Internet hospital. If 0 < *d* ≤ *d*_1_, effort is higher without it.

**Figure 3:**
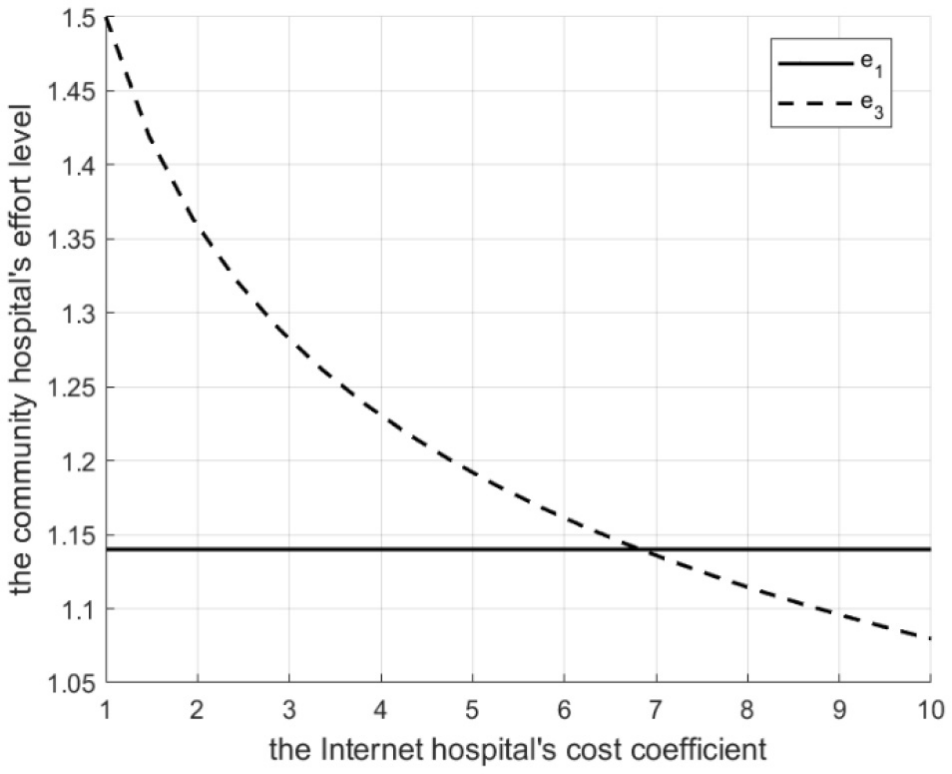
The Degree of effort to introduce a tightly integrated MA before and after the introduction of internet medical care.

Rationale and Simulation (Figure 3 *q*=10): When *d* is low, the Internet hospital operates at a lower cost and provides a high service level (γ), thereby reducing the community hospital's unit effort-related cost. To maximize profit (and reduce penalty costs which decrease with effort), the MA chooses a higher effort level (*e*_3_ > *e*_1_). When *d* is high, the Internet hospital's cost is high, the MA reduces γ to save costs, which increases the community hospital's unit effort-related cost, leading to lower optimal effort (*e*_3_<*e*_1_) (Figure 3 shows *e*_3_> *e*_1_ when *d* ≤ 6.8, where *e*_1_ and *e*_3_ represent the effort levels before and after implementing Internet-based healthcare in tightly integrated MA, respectively.).

### 4.2 Comparative profitability analysis in tightly integrated MA

Proposition 3: The profit margin in a tightly integrated MA (with vs. without Internet hospital) depends on Λ and *d*.

(1) There exists Λ_1_. If 0 < Λ ≤ Λ_1_, profit is higher with the Internet hospital. If Λ>Λ_1_, profit is higher without it.
(2) Λ_1_ decreases *d* as increases.

**Figure 4:**
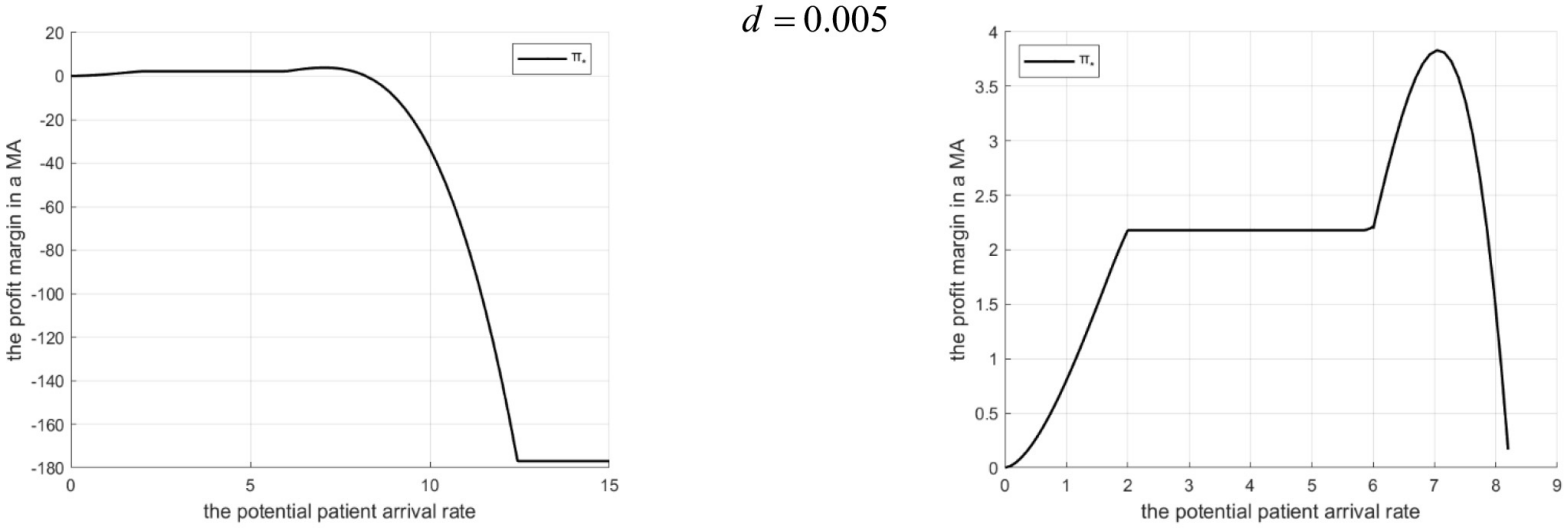
Profits of a tightly integrated MA before and after the introduction of internet medical care (*d* = 0.005).

**Figure 5:**
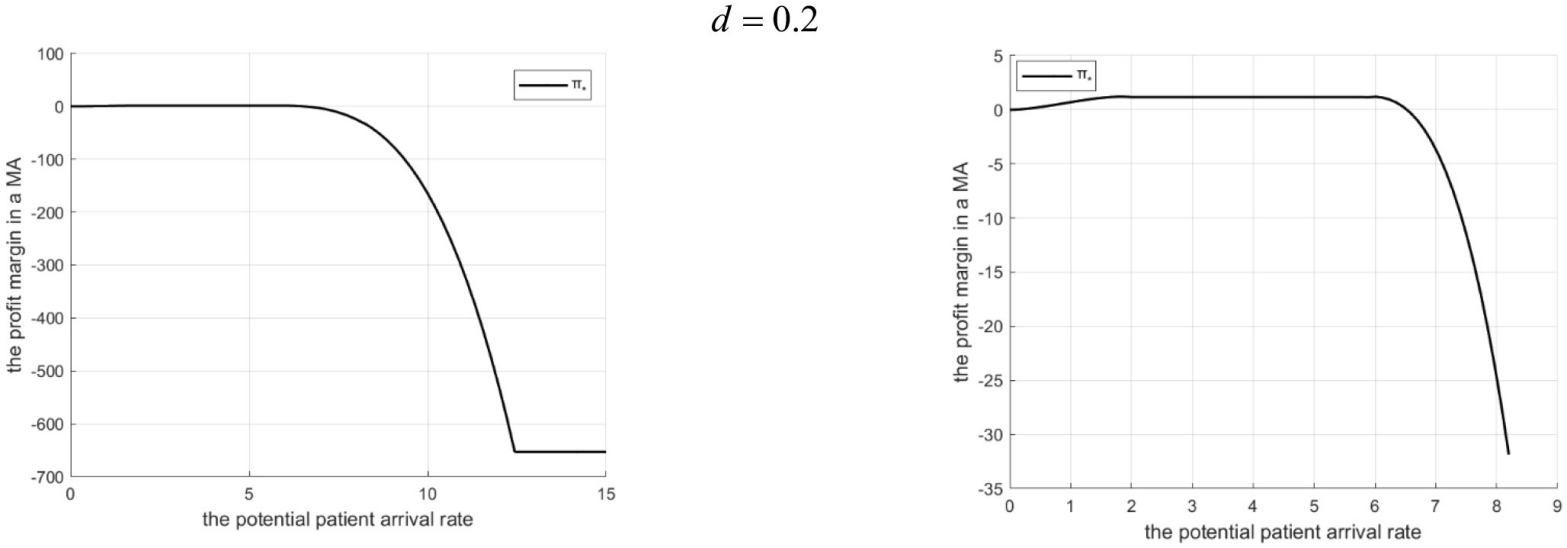
Profits of a tightly-integrated MA before and after the introduction of internet medical care (*d* = 0.2).

Rationale and Simulation (Figure 4 *d* = 0.005, Figure 5 *d* = 0.2): When Λ is low, introducing the Internet hospital increases referrals, boosting community hospital revenue. Scale effects cover Internet costs, and higher effort reduces penalties, increasing overall profit. When Λ is high, the increased referral threshold exceeds community capacity, leading to high penalty costs that damage MA profit, making the non-Internet scenario better. Λ_1_ (the threshold Λ where profits equalize) decreases with increasing *d* because higher Internet costs reduce the profitability range for the Internet hospital scenario (smaller referral increase, lower effort, higher potential penalty).

### 4.3 Comparative analysis of optimal decisions in loosely-integrated MA

Proposition 4: Referral quantity in a loosely-integrated MA depends on *d*, revenue sharing ratio β, and Λ.

**Figure 6:**
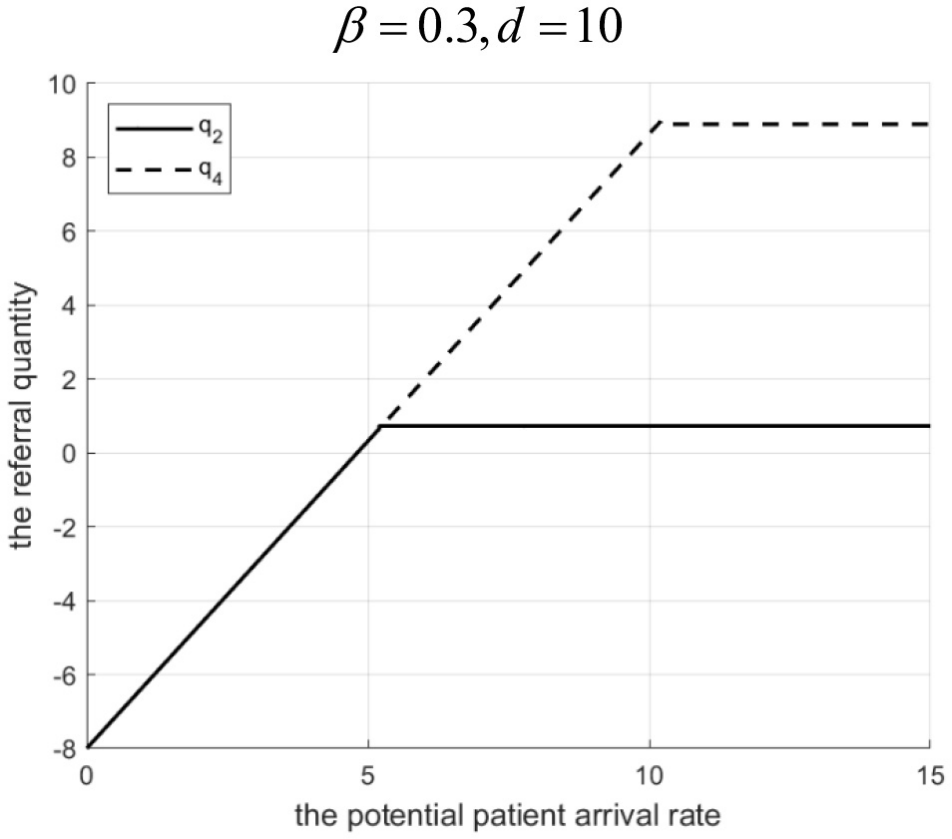
Referral quantity in a loosely integrated MA before and after the introduction of internet medical care (*β* = 0.3, *d* = 10 S).

**Figure 7:**
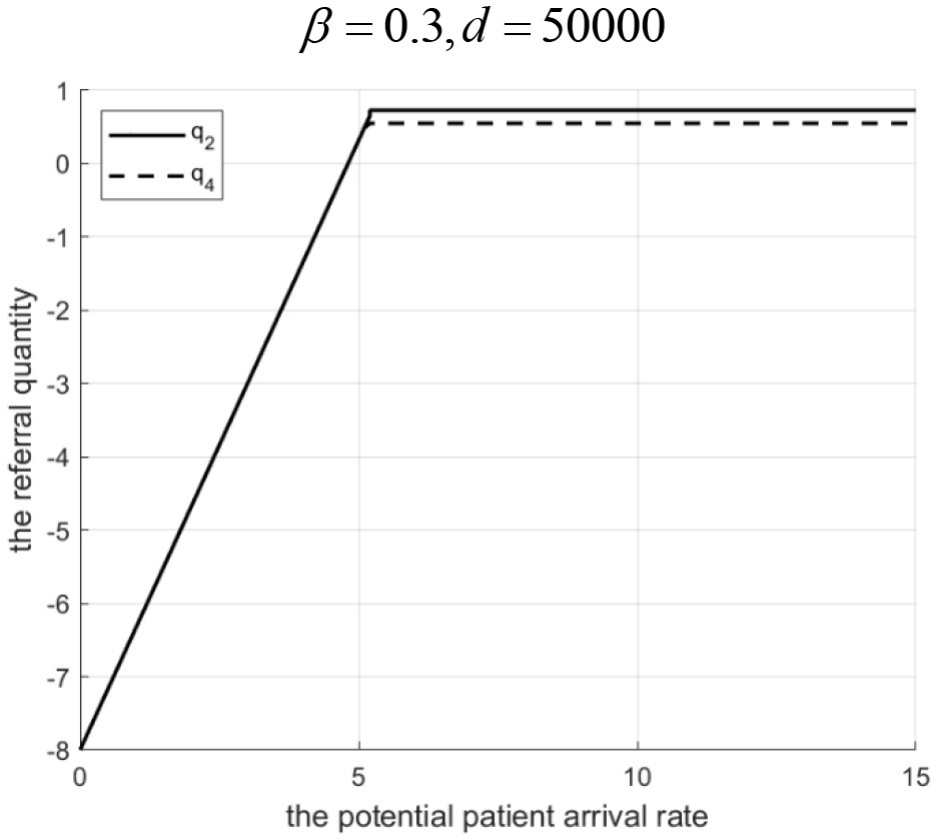
Referral quantity in a loosely integrated MA before and after the introduction of internet medical care (β = 0.3, *d* = 50, 000).

**Figure 8:**
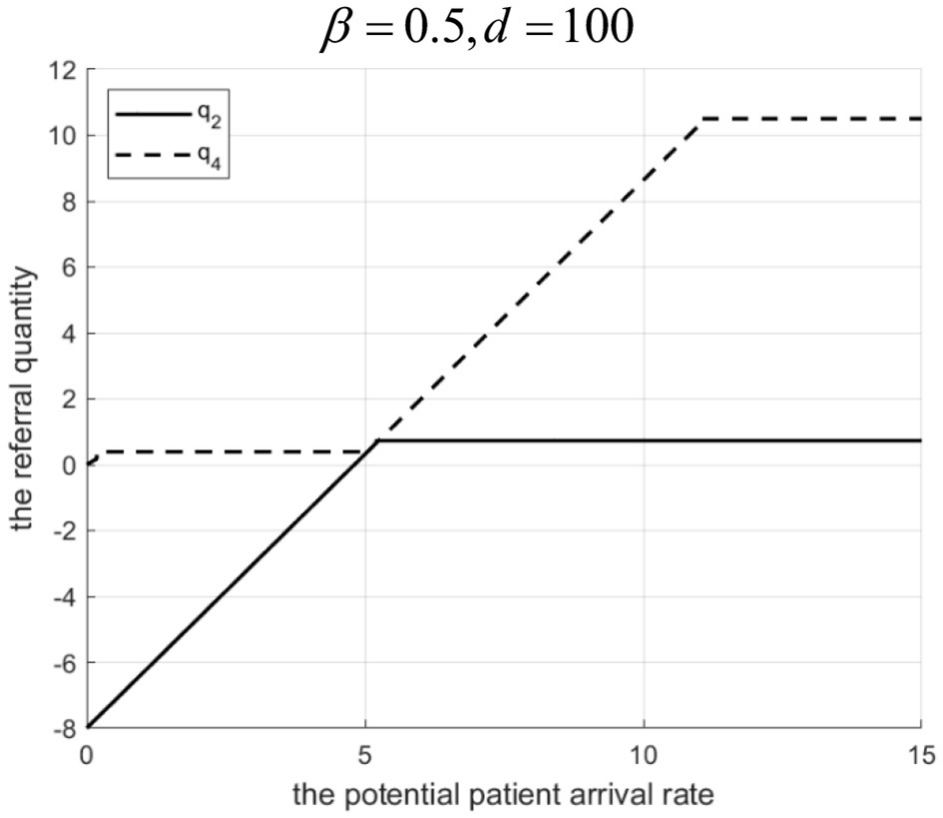
Referral quantity in a loosely integrated MA before and after the introduction of internet medical care (β = 0.5, *d* = 100).

**Figure 9:**
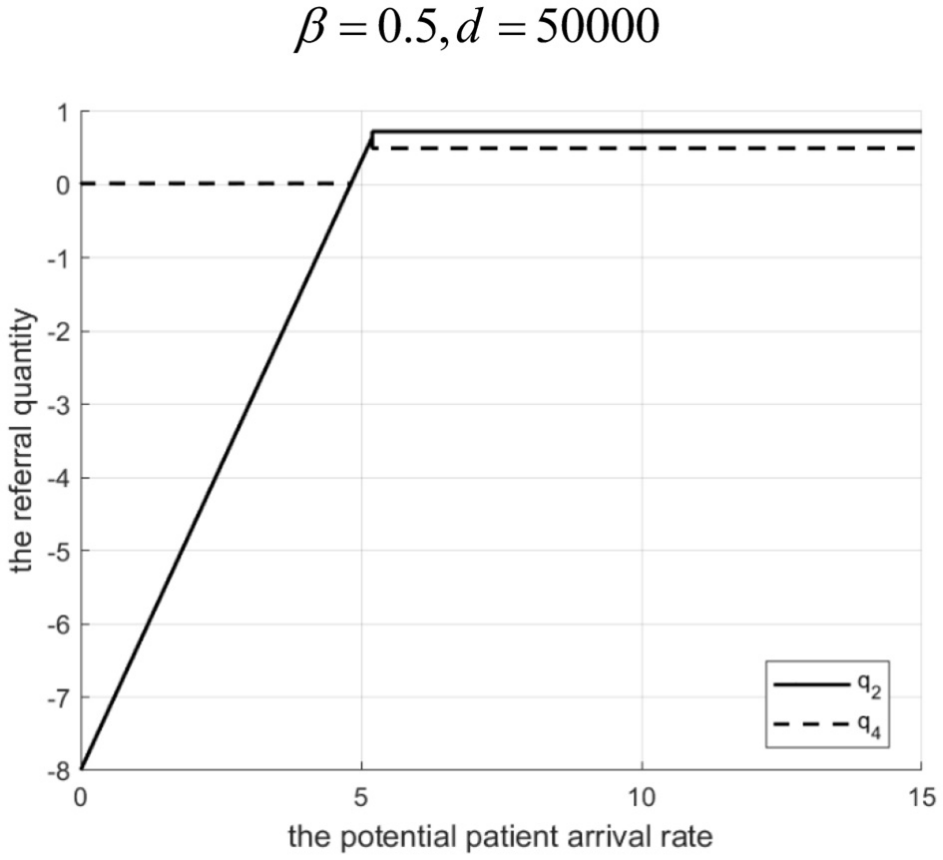
Referral quantity in a loosely integrated MA before and after the introduction of internet medical care (β = 0.5, *d* = 50, 000).

(1) If $0<\beta <\frac{g_{2}-C_{2H}}{g_{L}}$ (low sharing): For low Λ, Internet hospital cannot increase referrals. For high Λ, there exists *d*_1_. If 0 < *d*<*d*_1_, referrals are higher with the Internet hospital. If *d* ≥ *d*_1_, referrals are higher without it.
(2) If $\beta \geq \frac{g_{2}-C_{2H}}{g_{L}}$ (high sharing): For low Λ, Internet hospital can increase referrals. For high Λ, there exists *d*_2_. If 0 < *d* < *d*_2_, referrals are higher with the Internet hospital. If *d* ≥ *d*_2_, referrals are higher without it.
Rationale and Simulation (Figure 6 β=0.3, *d*=10; Figure 7 β = 0.3, *d*= 50,000; Figure 8 β = 0.5, *d* = 100; Figure 9 β= 0.5, *d*= 50,000):
Low β, low Λ: Tertiary hospital lacks incentive to refer as it gets a little share of community revenue.
Low β, high Λ: Tertiary hospital refers more. If *d* is low, Internet costs are covered by increased tertiary revenue (from reduced penalty share α*M*& β*g*_*L*_), supporting higher γ and referrals. If *d* is high, Internet cost > benefit, tertiary reduces γ and referrals.
High β, low Λ: Tertiary incentive exists (β*g*_*L*_ > *g*_2_). Even with low Λ, some referrals occur. Internet hospital (even if costly) can be used moderately to improve community services without high cost, facilitating referrals.
High β, high Λ: Tertiary wants high referrals. If *d* is low, Internet is cost-effective. If *d* is high, high γ is too costly, so tertiary reduces γ and referrals to balance costs. Let *q*_2_ and *q*_4_ denote the downward referral volumes before and after implementing Internet-based healthcare in loosely-integrated MA, respectively.

Proposition 5: For a given referral quantity, community hospital effort in a loosely integrated MA depends on *d*. There exists *d*_1_. If *d*_1_, effort is higher with the Internet hospital. If *d* > *d*_1_, effort is higher without it.

**Figure 10:**
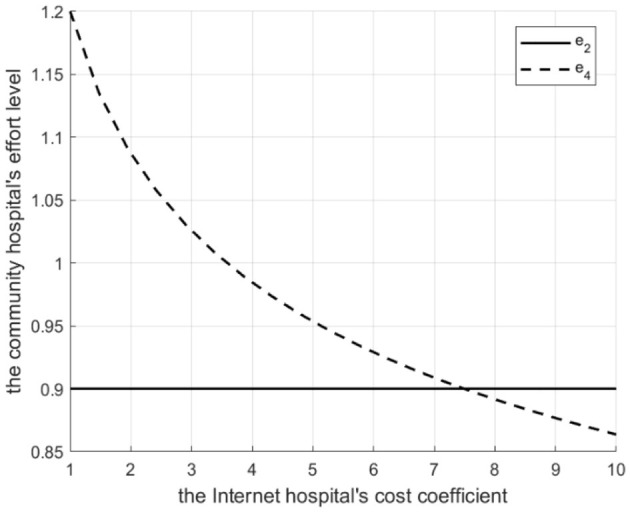
Degree of effort of loosely integrated MA before and after the introduction of internet medical care.

Rationale and Simulation (Figure 10 *q* = 10): Similar logic to Proposition 2, but driven by the tertiary hospital's decision on γ (to maximize its own profit by influencing penalty cost γ*M*) and the community hospital's response (balancing its revenue (1 − β)*g*_*L*_ against effort-related cost $\frac{C_{e}e^{2}}{\gamma }$ and penalty ((1 − α)*M*). Low *d*> high γ chosen by tertiary > lower unit effort-related cost for community > higher *e* chosen by community. High *d* > low > higher unit effort-related cost> lower *e*. *e*_2_ and *e*_4_ represent the effort levels before and after implementing Internet-based healthcare in loosely integrated medical MA, respectively.

### 4.4 Comparative profitability analysis in loosely integrated MA

Proposition 6: The tertiary hospital's profit margin depends on Λ, *d*, and β.

**Figure 11:**
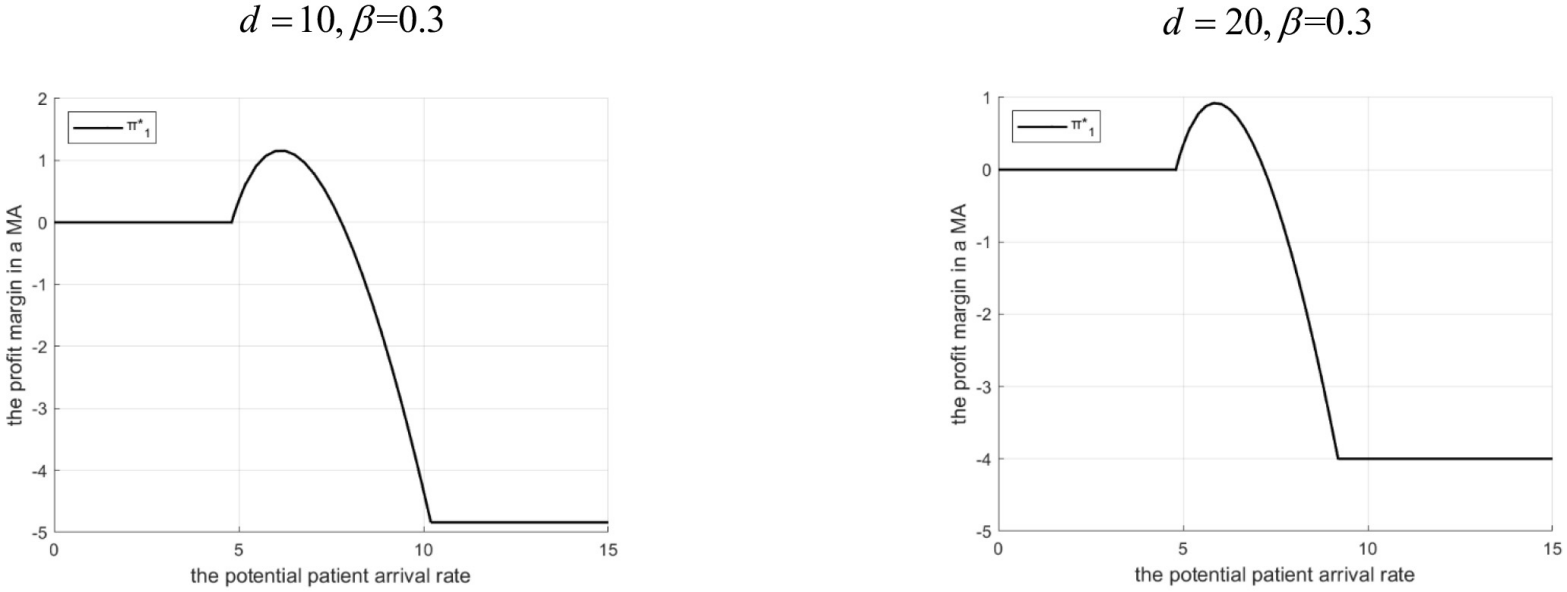
Profits of tertiary A-level hospitals before and after the introduction of internet medical care (β = 0.3).

**Figure 12:**
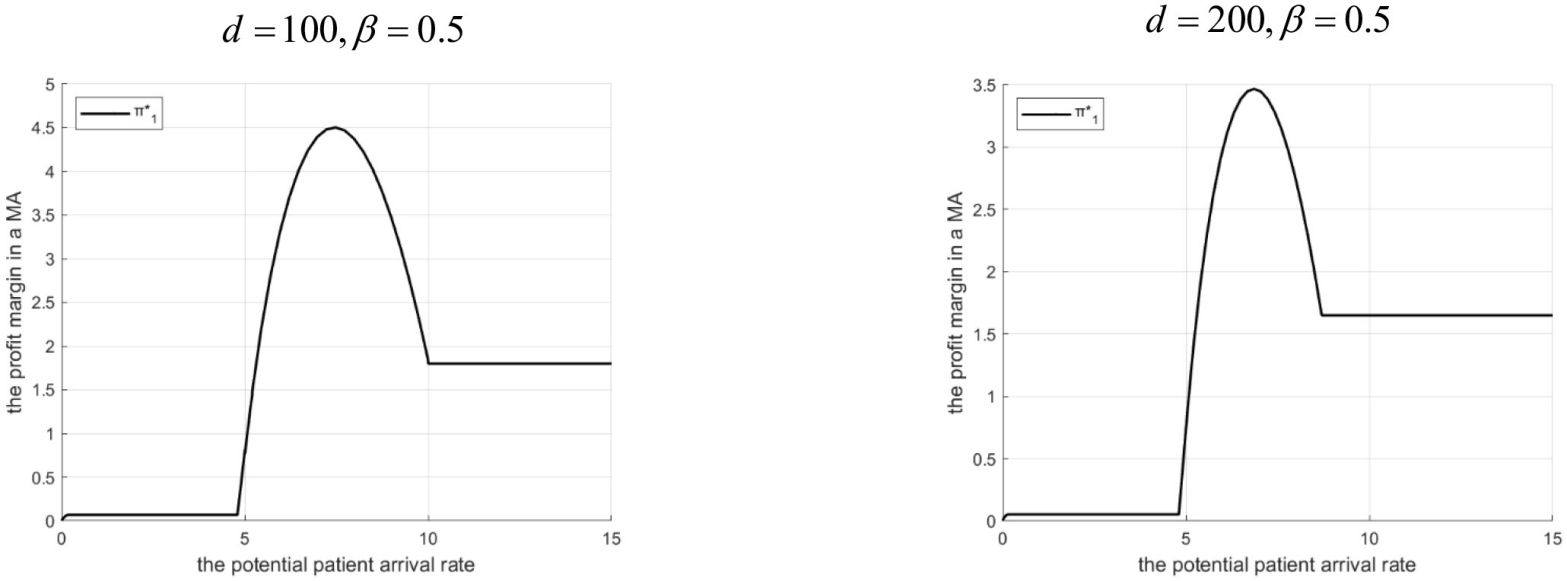
Profits of tertiary A-level hospitals before and after the introduction of internet medical care (β = 0.5).

(1) If $0<\beta <\frac{g_{2}-C_{2H}}{g_{L}}$: There exists Λ_2_. If 0 < Λ ≤ Λ_2_, profit is higher with the Internet hospital. If Λ > Λ_2_, profit is higher without it. Λ_2_ decreases as *d* increases.
(2) If $\beta \geq \frac{g_{2}-C_{2H}}{g_{L}}$: Profit is always higher with the Internet hospital. The profit advantage decreases as *d* increases.
Rationale and Simulation (Figure 11 β =0.3, *d*=10, *d*=20; Figure 12 β=0.5, *d*=100, *d*=200):
Low β, low Λ : Tertiary lacks referral incentive, Internet offers little benefit.
Low β, mid Λ : Internet helps increase referrals, brings revenue (β*g*_*L*_), reduces penalty share (α*M*)> profit increases.
Low β, high Λ : High referrals> high penalty cost share > profit erodes. Threshold Λ_2_ exists. Higher *d*> lower γ, smaller referral increase, less benefit > Λ_2_ decreases.
High β : The tertiary A-grade hospital always benefits from referrals. Internet facilitates this, adds revenue (β*g*_*L*_), and reduces penalty share. Even when *d* is high, it merely reduces the advantage rather than eliminating it—provided that the Internet hospital's cost remains lower than the benefit gained from increased or more efficient referrals.

Proposition 7: The community hospital's profit margin depends on Λ, *d*, and β.

**Figure 13:**
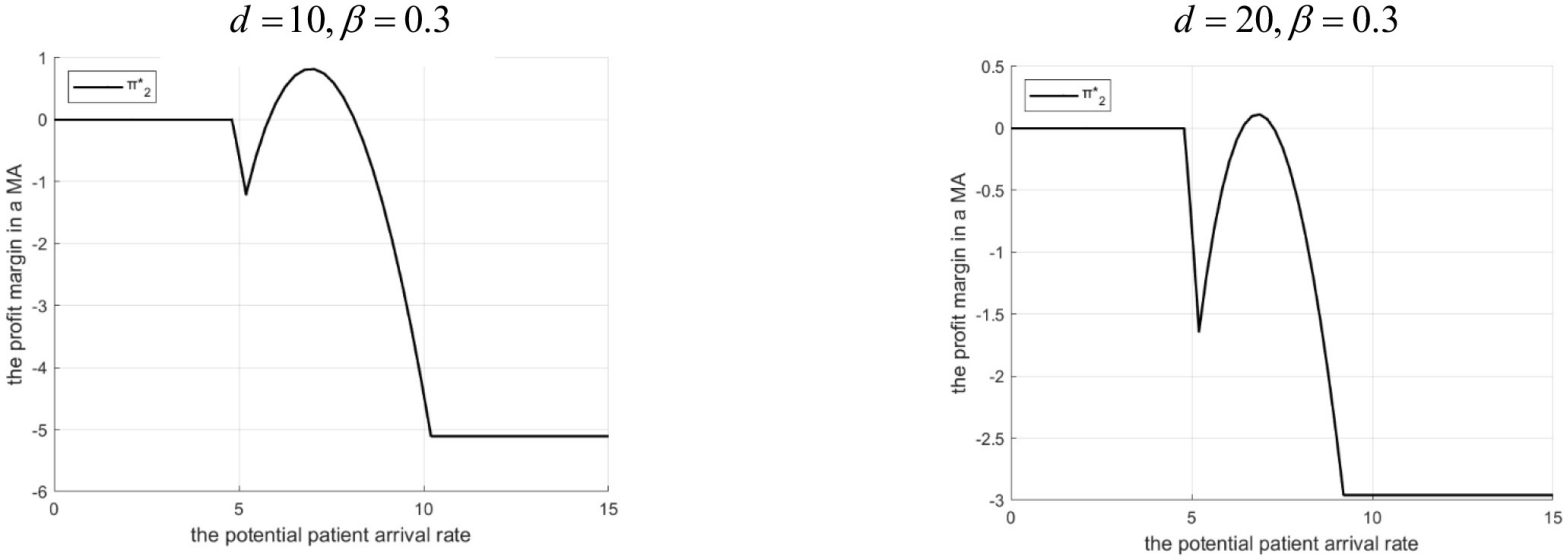
Profits of community hospitals before and after the introduction of internet medical care (β = 0.3).

**Figure 14:**
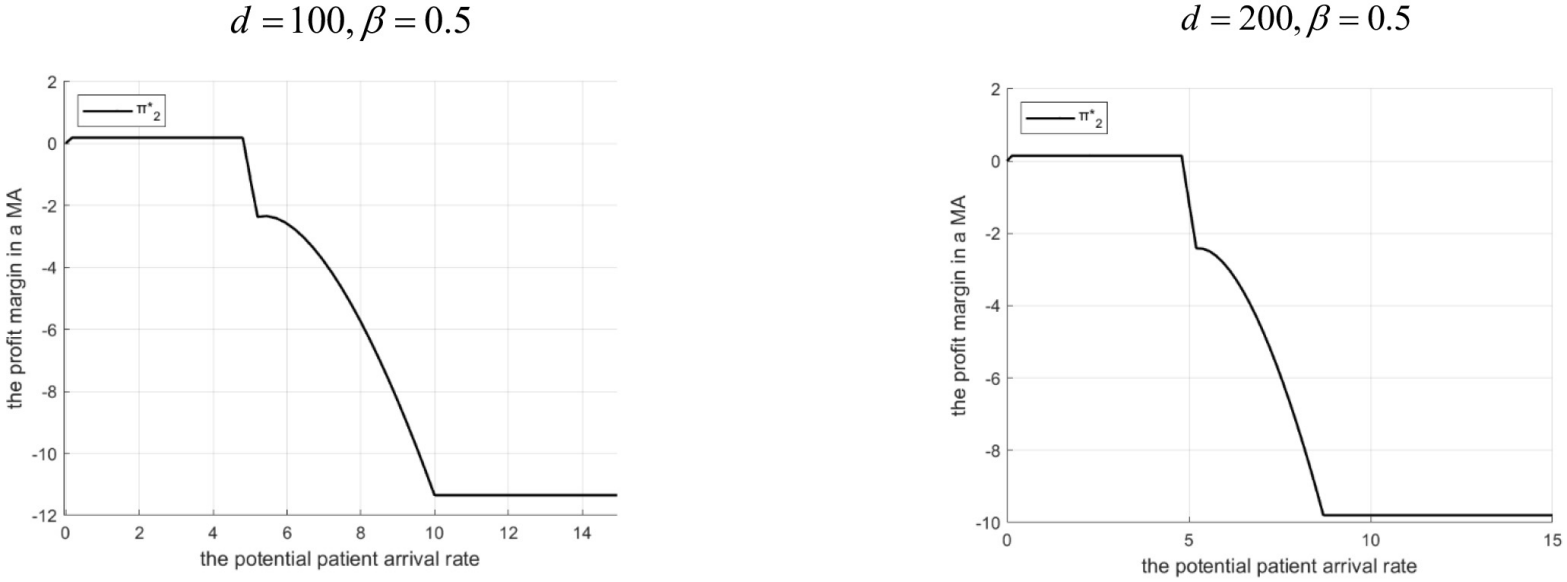
Profits of community hospitals before and after the introduction of internet medical care (β = 0.5).

(1) If $0<\beta <\frac{g_{2}-C_{2H}}{g_{L}}$: There exist Λ_3_ and Λ_4_ (Λ_3_ < Λ_4_). If 0 < Λ ≤ Λ_3_ or Λ ≥ Λ_4_, profit is not higher with the Internet hospital. If Λ_3_ < ΛΛ_4_, profit is higher with it. The interval[Λ_3_, Λ_4_] shrinks as *d* increases.
(2) If $\beta \geq \frac{g_{2}-C_{2H}}{g_{L}}$: There exists Λ_5_. If 0 < Λ ≤ Λ_5_, profit is higher with the Internet hospital. If Λ ≥ Λ_5_, profit is higher without it. The profit advantage (when with the Internet) increases with *d*.
Rationale & Simulation (Figure 13 β =0.3, *d*=10, *d*=20; Figure 14 β =0.5, *d*=100, *d*=200):
Low β: Community gets small share (1 − β)*g*_*L*_.
Low Λ: Tertiary doesn't refer much, Internet changes little for community profit.
Mid Λ (Λ_3_ < Λ ≤ Λ_4_): Tertiary refers more with Internet. Community gains revenue ((1 − β)*g*_*L*_) + benefits from higher γ (lower effort-related cost $\frac{C_{e}e^{2}}{\gamma }$) potentially offsetting penalty share (1 − α)*M* > profit increases.
High Λ (> Λ_4_): High referrals exceed capacity, high penalty share (1 − α)*M* > profit decreases. Higher *d* > smaller referral increase, less benefit, higher effort-related cost > interval [Λ_3_, Λ_4_]shrinks.
High β: Community gets large share (1 − β)*g*_*L*_.
Low Λ ( ≤ Λ_5_): Tertiary has an incentive to refer (due to its own high β). The Internet facilitates this. The community hospital benefits from increased revenue and reduced unit effort-related costs, resulting in higher overall profit.
High Λ (> Λ_5_): Tertiary refers heavily. Internet introduction leads to even higher referrals. However, the resulting rise in effort and penalty costs for the community hospital outweighs the additional revenue, leading to a decline in overall profit. Counterintuitively, profit advantage with Internet increases with *d* within the Λ ≤ range because higher *d* forces tertiary to use lower γ, reducing referrals slightly, which lessens the burden (effort/penalty) on the community hospital compared to a scenario with very low *d* and extremely high referrals.

## 5 Conclusion and outlook

This study addresses the suboptimal implementation of patient downward referrals within MAs, often driven by conflicting interests between tertiary A-grade hospitals and community hospitals as well as the latter's limited service capacity. It highlights the potential of Internet hospitals to promote referrals and enhance community hospital capabilities. By constructing queuing game models for tightly integrated and loosely integrated MAs, both before and after the introduction of hospital-owned Internet hospitals, this research investigates the conditions under which such supplementation is effectively promotes patient referrals across different MA types.

Key findings include:

Tightly integrated MAs: Referral quantity and effort levels increase with potential patient arrival rates (Λ). When the Internet hospital cost coefficient (*d*) is low, its introduction leads to higher (or equal) referral quantity and effort compared to scenarios without it; the opposite holds when *d*is high. Profitability is higher with the Internet hospital at lowerΛ, but higher without it at higher Λ, with the threshold arrival rate decreasing as *d* increases.

Loosely integrated MAs: Effort level changes follow a similar pattern to tightly integrated MAs based on *d*. Referral quantity and tertiary hospital profitability are highly sensitive to the revenue sharing ratio (β). Low β: Internet hospitals increase referrals only at high Λ and low *d*. Tertiary profit is higher with Internet hospitals only below a certain Λ threshold (Λ_2_), which decreases with *d*. High β : Internet hospitals can increase referrals even at low Λ. Tertiary profit is consistently higher with Internet hospitals, though the advantage diminishes with increasing *d*. Community hospital profit (Low β): Profitability is higher with Internet hospitals only within a specific intermediate range of Λ ([Λ_3_, Λ_4_]), which shrinks with increasing *d*. Community hospital profit (High β): Profitability is higher with Internet hospitals only below a certain Λ threshold (Λ_5_). The profit advantage increases with *d* within this range.

Based on these findings, several management implications emerge:

Strategic Introduction: Tightly integrated and loosely integrated MAs are advised to adopt hospital-owned Internet hospitals primarily under conditions of high potential patient inflow and low operational costs associated with Internet hospital implementation. Capacity Consideration: When implementing patient referrals, it is essential to account for the service capacity of community hospitals to avoid excessive referrals that may incur substantial penalty costs and compromise service quality. Revenue Sharing Design: Loosely integrated MAs should carefully calibrate the Internet healthcare revenue sharing ratio (β) to balance incentives for tertiary hospital referrals while safeguarding the financial sustainability of community hospitals. Policy Support: Governments may consider introducing supportive policies—such as providing technological subsidies to tertiary hospitals for establishing Internet hospitals—to encourage the adoption of digital health solutions and advance the implementation of the hierarchical diagnosis and treatment system.

This study assumes that the payment strategies and standards of the Medical Insurance Bureau remain static over time. Future research could incorporate the Medical Insurance Bureau into the game model to analyze the interplay among the bureau, MAs, and Internet hospitals, exploring how MA referral efficiency and Internet healthcare strategy choices might change under dynamic payment policies. Furthermore, this study focuses exclusively on hospital-owned Internet hospitals; Future work could broaden the scope to include third-party Internet hospitals by developing models that capture the interactions among MAs, Internet hospitals, and patients, thereby identifying the optimal choice of Internet hospital provider based on referral efficiency. Additionally, the current analysis assumes that tertiary hospitals maintain sole authority over downward referrals. Subsequent research could explore how patient transfer preferences affect Internet-based healthcare strategy decisions.

## Data Availability

The original contributions presented in the study are included in the article/supplementary material, further inquiries can be directed to the corresponding author.
